# Robustness of the aging effect of smiling against vertical facial orientation

**DOI:** 10.12688/f1000research.111126.1

**Published:** 2022-04-08

**Authors:** Naoto Yoshimura, Fumiya Yonemitsu, Kyoshiro Sasaki, Yuki Yamada

**Affiliations:** 1 Graduate School of Human-Environment Studies, Kyushu University, Fukuoka, Fukuoka, 819-0395, Japan; 2 Japan Society for the Promotion of Science, Chiyoda-ku, Tokyo, 102-0083, Japan; 3 Faculty of Letters, Chuo University, Hachioji, Tokyo, 192-1393, Japan; 4 Faculty of Informatics, Kansai University, Takatsuki, Osaka, 569-1095, Japan; 5 Faculty of Arts and Science, Kyushu University, Fukuoka, Fukuoka, 819-0395, Japan

**Keywords:** Facial expression, Age estimation, Face inversion

## Abstract

**Background:** Previous studies have shown that the association between smiling and youth is a misconception; smiling faces have been estimated to be older than neutral faces. Previous studies have indicated that this aging effect of smiling (AES) is due to eye wrinkles caused by the facial action of smiling. However, whether holistic processing for facial expressions is involved in AES has not been investigated. The present study aimed to clarify these issues.

**Methods: **Participants were recruited to participate in an online experiment that had a 3 (facial expression: smiling/neutral/surprised) × 2 (facial orientation: upright/inverted) mixed design. Participants were presented with an upright or inverted face for each expression (neutral, smiling, and surprised) and were asked to estimate the individual’s age.

**Results: **In total, 104 participants were included in the analysis. The results show that smiling faces were estimated to be older than neutral faces, whereas there was no significant difference between upright and inverted faces.

**Conclusions: **Our findings suggest that direct age estimation is not dependent on holistic processing.

## Introduction

The face is a valuable source of information for social communication, and humans have developed specific processing methods for others’ faces. For example, they can perceive the identity, gender, age, ethnicity, attractiveness, and emotions of others from their faces (e.g., Bruce & Young, 1986; Ganel & Goshen-Gottstein, 2002). These multiple abilities for face processing highlights that the face is processed holistically in contrast to other visual stimuli. Therefore, we are unable to identify others’ faces and judge facial expressions when holistic processing is inhibited (e.g., Rakover, 2013). This holistic processing of the face creates complex interactions between multiple factors, such as the interaction between emotion and gender ( Atkinson *et al*., 2005).

Age is among the crucial information obtained from the face. We generally estimate a person’s age from their faces and accordingly change our attitude and manner of speaking ( Ryan *et al*., 1986). Among the many information dimensions that can easily be extracted from a face, age is considered the primary dimension ( George & Hole, 1998). Thus, accurate age identification is crucial in determining social roles and facilitating social interaction. However, various factors distort age perception (see Moyse, 2014). Previous studies have specifically focused on the effects of gender and race on perceived age ( Dehon & Brédart, 2001; Nkengne *et al*., 2008).

Interestingly, several studies have reported that humans have a counterintuitive bias regarding age. We associate smiling with youth, that is, it is generally believed that when people see a smiling person, they feel that person is younger. Indeed, previous research has provided evidence that individuals with a smile appear younger than those with other facial expressions ( Hass *et al*., 2016; Voelkle *et al*., 2012). However, contrary to the commonly held association between smiling and youth, Ganel (2015) showed that a smiling face is estimated to be older than a neutral one. The aging effect of smiling (AES) is attributed to wrinkles around the eyes caused by smiling ( Ganel, 2015; Ganel & Goodale, 2021). In contrast, when participants were asked to retrospectively estimate the mean age of several faces (i.e., face group), they estimated that the smiling face group was younger than the neutral face group ( Ganel & Goodale, 2018). These studies indicate that the effect of emotional expressions on age estimation depends on the method of estimation (i.e., directly or retrospectively).

Recently, our study showed that AES was consistently confirmed regardless of the stimulus or participants’ race or culture ( Yoshimura *et al*., 2021). Specifically, smiling faces were estimated to be older than neutral faces for both Swedes and the Japanese. In contrast, participants in both countries estimated the smiling faces to be younger when estimating the age retrospectively. These results suggest that AES is robust across cultures, although the direction of this effect changes depending on the task.

The AES mechanism, however, remains unclear. What can be assumed is that AES is associated with some type of characteristic information of face perception or emotion processing that accompanies changes in facial expressions. Age is considered a relatively primary piece of information extracted from the face ( Bruce & Young, 1986), and previous research suggests that age perception may be based on facial surface or shape information ( George & Hole, 2000). However, other studies have suggested that facial expression processing relies on holistic processing ( Maurer *et al*., 2002; Tanaka & Sengco, 1997). As shown in previous research, facial expressions interact with other dimensions such as identity and trustworthiness ( Engell *et al*., 2010; Ganel & Goshen-Gottstein, 2002). When the holistic processing of facial expressions is inhibited, observers’ perceptual fields are constricted and facial features are processed sequentially and independently ( Rossion, 2009). In such cases, inverted smiling faces could be evaluated as older than upright smiling faces.

To extend the AES findings, the present study examined how the holistic processing of facial expressions could be involved in AES. Here, we used inverted faces in the experiment because they inhibit the holistic processing of facial expressions while maintaining visual information (e.g., Rakover, 2013). Specifically, we divided the participants into two groups: one observing upright faces, and the other observing inverted faces. They were asked to estimate the ages of the smiling, neutral, and surprised faces. The estimated age for each facial expression obtained was then compared between the groups. Even if AES was boosted with an inverted smiling face, we could not rule out the possibility that inversion itself has the effect of making the facial expression stimulus older. Hence, we set a surprised face (i.e., neutral expression) as the control condition. If the enhancement of AES was due to the prioritization of local information processing for the smiling expression as holistic processing was suppressed, there would not be a significant difference between facial orientations in the surprised face.

## Method

### Study design and participants

This study employed a mixed factorial design. The participants were recruited through the online survey platform, Yahoo! Crowdsourcing. The target age was 15-35 years old to address potential sources of bias caused by unexpected deviations in the age of the respondents. The survey was published on the platform (survey period: November 24-25, 2021) and participants could select to participate in the survey for a minimal compensation of 10 “PayPay bonus rights” (electronic money). We determined the sample size to be *N* = 100 because Yahoo! Crowdsourcing has specifications for recruiting participants in units of 50; thus, we recruited 50 participants per facial orientation group. Participants were recruited separately for tasks in which upright or inverted faces were presented, and the study’s purpose was not disclosed to the participants.

### Ethical approval and consent to participate

The experiment was conducted in accordance with the principles of the Declaration of Helsinki. The ethics committee of Kyushu University approved the study protocol (approval date: July 27, 2021; approval number: 2021-013). Completion of the experiment was taken as consent to participate from participants. Participants had the right to withdraw from the experiment at any time without providing a reason. It was also explained to them that their responses would not be tied to them personally.

### Facial stimuli

The Japanese facial stimuli consisted of head-and-shoulder photos of 30 women and 30 men with smiling, neutral, and surprised expressions from the ATR Facial Expression Image Database (DB99) (ATR-Promotions, Kyoto, Japan; 2562 photos; Ogawa & Oda, 1998; *M*_age_ = 21.1 years, ranging from 20 to 24 years). The face image database systematically contains the faces of male and female individuals and their three facial expressions. The first 30 images from each list of the faces were used as the facial stimuli for the present study, thus a total of 180 images were selected (2 genders × 3 facial expressions × 30 individuals). Japanese facial photos were adjusted to 7 × 9 cm and divided into three sets for each emotional expression (smiling, neutral, and surprised sets), with each set consisting of 60 photos. Next, we prepared six counterbalanced sets of 60 photos by extracting 20 photos from each emotional expression set. This was done to avoid presenting the same individuals repeatedly with different facial expressions. Therefore, the participants were randomly assigned to one of the six counterbalanced sets.

### Procedure

The experiments were conducted online, and the procedures were controlled using jsPsych (Version 6.3.1; de Leeuw & Motz, 2016). In addition, the Cognition platform was used for data collection. In each trial, participants were presented with a smiling, neutral, or surprised face. They were then asked to estimate the age of each facial stimulus and enter their estimated age in a text box. To detect satisficers ( Maniaci & Rogge, 2014), the directed questions scale (DQS) (answer: “9 years old”) was also set on the 30th trial of the task. In this DQS trial, we also presented a beast-man ^1^ with the same composition as the other stimuli.

### Statistical analysis

All analyses were conducted using RStudio (Version 1.4.1717; RStudio Team, 2021) and R (Version 4.1.1; R Core Team, 2020). A two-way mixed analysis of variance (ANOVA) was performed to examine the differences in the estimated age of the facial expressions of each face group. Subsequently, a Scheffé multiple comparison test was also performed to compare the difference in each pair. The alpha level of statistical significance was set at 0.05.

## Results

From the internet protocol addresses collected in the experiment, we checked whether any individuals participated in both the upright and inverted face conditions. In total, 104 Japanese people (52 people per group) were recruited to participate in the online experiment (51 males, 51 females, and two non-respondents; *M*_age_ = 29.56, *SD* = 7.04). Duplicate data were excluded from the analysis because we could not confirm whether they were answered by different individuals. We also excluded data from participants who answered the DQS incorrectly. The final number of valid data used in the analysis was 98 (we excluded data from six participants) ( Yoshimura, 2022). We also removed trials where the estimated ages were outside ± 2.5 SD from the participants’ mean in each condition.

**Figure 1.**
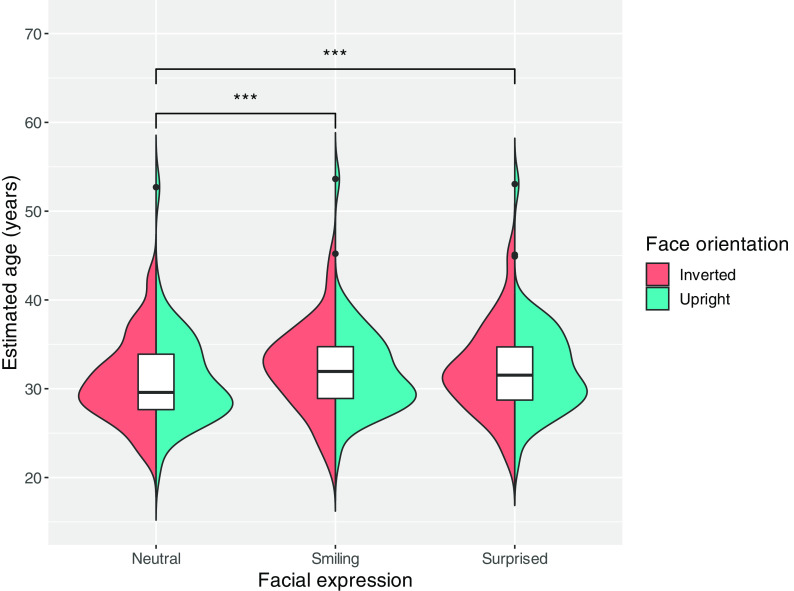
Violin and box plots showing the distribution of mean estimated age for each expression by facial orientation. *p* < 0.001 = ***.

Figure 1 shows the distribution of the mean estimated age for each expression in the upright and inverted face conditions. We conducted a two-way mixed ANOVA with facial expression (smiling, neutral, or surprised) as the within-subjects factors and facial orientation (upright or inverted) as the between-subjects factors. The results revealed a main effect of facial expression ( *F*(2, 189) = 23.11, *p* < .001, η ^2^_G_ = .01). However, the main effect of facial orientation ( *F*(1, 96) < 0.000, *p* = .98, η ^2^_G_ < .001) and the interaction between facial expression and facial orientation ( *F*(2, 189) = 1.33, *p* = .27, η ^2^_G_ = .01) were not significant. Based on the main effect of facial expression, we also conducted a Scheffé multiple comparison test of the facial expressions. The results showed that participants estimated smiling faces to be significantly older than neutral faces ( *t*(96) = 5.52, *p* < .001, *d* = 0.56). In addition, the results also showed that they estimated surprised faces to be significantly older than neutral faces ( *t*(96) = 6.40, *p* < .001, *d* = 0.65).

We performed an equivalence test ( Lakens *et al*., 2018) for facial orientation as a post-hoc and exploratory analysis. We set equivalence bounds to ± 0.5, as the medium effect size (i.e., Cohen's *d*). The results showed that the mean estimated ages in the upright and inverted conditions were significantly equivalent ( *t*(95.34) = 2.453, *p* < 0.01).

## Discussion

The present study aimed to examine how holistic processing of facial expressions contributes to AES. In the experiment, we asked two groups of participants (given upright or inverted faces) to estimate the age of each facial expression; we then compared the estimated ages. The results showed that smiling faces were estimated to be older than neutral faces, indicating that AES was replicated. However, there was no significant difference in the estimated age between the upright and inverted conditions. More importantly, AES was confirmed even when inverted faces were presented. We predicted that inverted smiling faces would be evaluated as even older than upright faces, because holistic processing was inhibited in favor of local processing. However, the analysis revealed no significant effect on facial orientation. Furthermore, we conducted an equivalence test for facial orientation as an exploratory and post-hoc analysis and found that the mean estimated ages in both upright and inverted conditions were significantly equivalent. Thus, the results suggest that direct age estimation is insensitive to holistic processing.

Given the results of this experiment, we assume that the holistic processing of emotional expressions is not sufficient to significantly modulate AES. Another unexpected result was an increase in the estimated age of the surprised faces. This suggests that changes in face shape, rather than the association between smiling and youthfulness, affected AES. The results highlight the possibility that AES is processed based on the perceptual analysis of facial features, that is, the structural encoding stage ( Calder & Young, 2005). A previous study also reported no decrease in the accuracy of age estimation, even when participants estimated the age of inverted, negation, or blurred face stimuli ( George & Hole, 2000). Recent studies have also reported that AES is affected by changes in the skin surface and other facial parts (e.g., wrinkles around the eye region) over the lifespan ( Ganel & Goodale, 2021). The results of our study are consistent with these findings. Considering that age is one of the social characteristics critical to encoding the identity of others, the results of this study seem reasonable.

Another possible factor is that the attractiveness of facial parts may affect age estimation. Some previous studies have reported that masked faces were more attractive or vice versa ( Hies & Lewis, 2022; Miyazaki & Kawahara, 2016; Patel *et al*., 2020). The fact that age could be estimated only for the upper half of the face and that age estimation did not depend on holistic processing leads to speculate the potential involvement of attractiveness. Specifically, the change in shape due to facial expressions may have reduced the attractiveness of the parts, thereby altering the apparent age. Therefore, the attractiveness of facial parts is also worth considering in further studies, such as whether it is indeed involved and, if so, what the causal mechanism entails.

The findings of the present study indicate that the association between smiling and youth is a misconception, and that direct age estimation is based on more primary and local facial features. However, it remains unclear why smiling faces are rated as younger in memory-based age estimation. Age estimation for memory representations of faces may be processed through different mechanisms than that for perceptual representations of faces in the process of dissociated facial processing (e.g., Weigelt *et al*., 2014), and such a bias in opposite directions in memory and perception for identical stimuli has been observed in spatial processing ( Yamada *et al*., 2011). Future research should further examine these questions.

### Limitations

The present study did not address the cross-cultural validity. A previous study compared the differences between Western Caucasians and East Asians in eye movements to inverted faces ( Rodger *et al*., 2010). This study reported cultural differences in the fixation area to the face, even for inverted faces. Given the results, it should be noted that the results of this study are generalizable only to Japanese participants estimating the age of Japanese faces.

## Data availability

### Underlying data

Open Science Framework: Age Estimation and Face Inversion. https://doi.org/10.17605/OSF.IO/7P25C ( Yoshimura, 2022).

This project contains the following underlying data:

- AgeEstimation_dataset.csv (The dataset)
- Description of Dataset.txt

Data are available under the terms of the Creative Commons Attribution 4.0 International license (CC-BY 4.0).

## Competing interests

No competing interests were disclosed.

## Grant information

This study was supported by Japan Society for the Promotion of Science KAKENHI Grant Number JP19J21874 to N. Y., 19K14482 to K.S., JP21J01431 to F.Y., JP16H03079, JP17H00875, JP18K12015, JP20H04581 to Y.Y., and JP21H03784 to K.S. and Y.Y.

## Footnotes

^1^
Image of a cat’s face superimposed on a human face.
