# Supplementary material for: Robustness of the aging effect of smiling against vertical facial orientation
Source: F1000Res. 2022 Jul 4;11:404. Originally published 2022 Apr 8. [Version 3] doi: 10.12688/f1000research.111126.3 (PMC9247519; doi:10.12688/f1000research.111126.3)
Supplement: Peer review history

# Peer review: Reviewer response for version 3

**DOI:** 10.5256/f1000research.135719.r143194

**Published:** 2022-07-05

**Authors:** Tzvi Ganel

**Affiliations:** 1 Department of Psychology, Ben-Gurion University of the Negev, Beer-Sheva, Israel

Approved.

Is the work clearly and accurately presented and does it cite the current literature?

Partly

If applicable, is the statistical analysis and its interpretation appropriate?

Yes

Are all the source data underlying the results available to ensure full reproducibility?

Yes

Is the study design appropriate and is the work technically sound?

Yes

Are the conclusions drawn adequately supported by the results?

Partly

Are sufficient details of methods and analysis provided to allow replication by others?

Yes

Reviewer Expertise:

Perception and action; Visual psychophysics; Face perception

I confirm that I have read this submission and believe that I have an appropriate level of expertise to confirm that it is of an acceptable scientific standard.

# Peer review: Reviewer response for version 2

**DOI:** 10.5256/f1000research.135239.r141470

**Published:** 2022-06-30

**Authors:** Tzvi Ganel

**Affiliations:** 1 Department of Psychology, Ben-Gurion University of the Negev, Beer-Sheva, Israel

The authors did a very good effort in their revision and have now responded to my comments. I have no further comments.

Is the work clearly and accurately presented and does it cite the current literature?

Partly

If applicable, is the statistical analysis and its interpretation appropriate?

Yes

Are all the source data underlying the results available to ensure full reproducibility?

Yes

Is the study design appropriate and is the work technically sound?

Yes

Are the conclusions drawn adequately supported by the results?

Partly

Are sufficient details of methods and analysis provided to allow replication by others?

Yes

Reviewer Expertise:

Perception and action; Visual psychophysics; Face perception

I confirm that I have read this submission and believe that I have an appropriate level of expertise to confirm that it is of an acceptable scientific standard.

# Response

**Published:** 2022-06-30

**Authors:** Naoto Yoshimura

**Affiliations:** Kyushu University, Japan

Thank you for your favorable evaluation of our revised manuscript. Based on the comment of another reviewer, we corrected only the typography.

# Peer review: Reviewer response for version 2

**DOI:** 10.5256/f1000research.135239.r141469

**Published:** 2022-06-29

**Authors:** Michiko Asano

**Affiliations:** 1 Department of Psychology, Graduate School of Humanities and Sociology, The University of Tokyo, Tokyo, Japan

I found the paper improved in clarity, and the authors adequately addressed each of my points.

 [Typo?]

 The fourth sentence of the first paragraph of the introduction, “Therefore, we cannot to identify…” -> “Therefore, we cannot identify…”?

Is the work clearly and accurately presented and does it cite the current literature?

Partly

If applicable, is the statistical analysis and its interpretation appropriate?

Yes

Are all the source data underlying the results available to ensure full reproducibility?

Yes

Is the study design appropriate and is the work technically sound?

Yes

Are the conclusions drawn adequately supported by the results?

Partly

Are sufficient details of methods and analysis provided to allow replication by others?

Yes

Reviewer Expertise:

cognitive psychology

I confirm that I have read this submission and believe that I have an appropriate level of expertise to confirm that it is of an acceptable scientific standard.

# Response

**Published:** 2022-06-30

**Authors:** Naoto Yoshimura

**Affiliations:** Kyushu University, Japan

Thank you for your positive feedback on the revised manuscript. It was also very helpful that you found the typographical error. We have corrected that typo.

# Peer review: Reviewer response for version 1

**DOI:** 10.5256/f1000research.122814.r136662

**Published:** 2022-06-13

**Authors:** Michiko Asano

**Affiliations:** 1 Department of Psychology, Graduate School of Humanities and Sociology, The University of Tokyo, Tokyo, Japan

This study investigated the contribution of holistic face processing on the aging effect of smiling (AES) in direct age estimation of faces. The authors conducted an experiment in which participants were presented with either upright or inverted faces and asked to estimate the individuals’ age. There were three conditions involving the expressions of the faces: smiling, surprised, and neutral. The results showed that AES was observed regardless of the face orientations, suggesting that holistic processing of facial processing does not contribute to the occurrence of AES. The results also showed that surprised faces, as well as smiling faces, were estimated to be older than neutral faces.

 The work is technically sound, and the topic and results would be of interest to a broad audience. However, I have some concerns, both major and minor, which I address below.

**Major concerns:**

 (1) The flow of logic regarding the hypothesis is difficult to understand and needs to be improved.

 In the fifth paragraph of the introduction, the authors write *“… and previous research suggests that age perception may be based on facial surface or shape information (George & Hole, 2000). However, other studies have suggested that facial expression processing relies on holistic processing (Maurer et al., 2002; Tanaka & Sengco, 1997). As shown in previous research, facial expressions interact with other dimensions such as identity and trustworthiness (Engell et al., 2010; Ganel & Goshen-Gottstein, 2002). When the holistic processing of facial expressions is inhibited, observers’ perceptual fields are constricted and facial features are processed sequentially and independently (Rossion, 2009). In such cases, inverted smiling faces could be evaluated as older than upright smiling faces”.*

 Overall, the text in this paragraph seems to be a bit short on explanations and hard to follow. If I understand correctly, I think the authors are trying to say the following here:

 (a) Some previous studies suggest that age estimation relies on local features of faces.

 (b) Some previous studies suggest that facial expression processing relies on the holistic processing of the faces.

 (c) Holistic processing is inhibited when faces are inverted.

 (d) Disruption of holistic face processing would lead to a greater impact of the local facial feature processing on age estimation. When holistic processing is inhibited, facial expression processing and the related complex cognitive processing would also be inhibited. It has also been reported that facial features are processed sequentially and independently when holistic processing is inhibited.

 (e) AES may be caused by a local facial feature, that is, wrinkles around the eyes caused by smiling, not by the facial expression of smiling per se.

 (f) Collectively, due to the relatively heightened impact of local feature processing in inverted faces, inverted smiling faces may be evaluated as older than upright smiling faces.

 However, the explanation about (e) is lacking from the current text, which makes it difficult to understand the logic here.

 I also found it confusing that the authors predict/interpret the results of their experiment in terms of “whether holistic processing is involved in AES” in some parts of the manuscript and “whether the relatively heightened impact of local feature processing in the inverted face condition leads to greater AES” in other parts without a clear and explicit distinction between the two.

 (2) It is not clear to me why the authors set the surprised face condition. The authors write, “ *Hence, we set a surprised face (i.e., neutral expression) as the control condition.*” in the last paragraph of the introduction. I do not understand this sentence because a surprised face cannot be regarded as an emotionally neutral face and, in addition, the authors set the neutral expression condition besides the surprise expression condition.

 I assume that the authors wanted to use a facial stimulus with an expression that was not a smile. If so, the authors should describe the difference between smiling and surprised (and neutral) faces in terms of the local features.

**Minor concerns and comments:**

 (3) Not only the smiling faces but also the surprised faces were estimated to be older in the current experiment. I suspect that this may be due to the wrinkles on the forehead and nasolabial folds when a surprised expression is generated.

 (4) The last sentence of the third paragraph of the introduction, “ *These studies indicate that the effect of emotional expressions on age estimation depends on the method of estimation (i.e., directly or retrospectively).*”:

 Does the term “AES” refer only to the phenomenon that smiling faces are estimated to be older than neutral faces in direct age estimations, or does it also refer to the phenomenon that smiling face group is retrospectively estimated to be younger? I got confused to read the sentence above.

 (5) The second to last sentence of the fifth paragraph of the introduction, “ *perceptual fields*”:

 This term requires a brief explanation.

 (6) Figure 1: The authors should explain what the black dots in this figure are.

 (7) The last sentence of the second paragraph of the discussion, “ *Considering that age is one of the social characteristics critical to encoding the identity of others, the results of this study seem reasonable.*”:

 I am having difficulty figuring out what they mean here. Specifically to which results are they referring?

 (8) The first sentence of the fourth paragraph of the discussion, “ *The findings of the present study indicate that the association between smiling and youth is a misconception, …*”:

 Given that the authors, as they state, did not investigate whether smiling faces are rated as younger in memory-based age estimation, the word “misconception” may be too strong and should be toned down.

Is the work clearly and accurately presented and does it cite the current literature?

Partly

If applicable, is the statistical analysis and its interpretation appropriate?

Yes

Are all the source data underlying the results available to ensure full reproducibility?

Yes

Is the study design appropriate and is the work technically sound?

Yes

Are the conclusions drawn adequately supported by the results?

Partly

Are sufficient details of methods and analysis provided to allow replication by others?

Yes

Reviewer Expertise:

cognitive psychology

I confirm that I have read this submission and believe that I have an appropriate level of expertise to confirm that it is of an acceptable scientific standard, however I have significant reservations, as outlined above.

# Response

**Published:** 2022-06-17

**Authors:** Naoto Yoshimura

**Affiliations:** Kyushu University, Japan

*This study investigated the contribution of holistic face processing on the aging effect of smiling (AES) in direct age estimation of faces. The authors conducted an experiment in which participants were presented with either upright or inverted faces and asked to estimate the individuals’ age. There were three conditions involving the expressions of the faces: smiling, surprised, and neutral. The results showed that AES was observed regardless of the face orientations, suggesting that holistic processing of facial processing does not contribute to the occurrence of AES. The results also showed that surprised faces, as well as smiling faces, were estimated to be older than neutral faces.*

*The work is technically sound, and the topic and results would be of interest to a broad audience. However, I have some concerns, both major and minor, which I address below.*

**Reply: **Thank you for taking the time and effort to check our manuscript. We appreciate your helpful comment. Individual replies to your comments are provided below.

*Major concerns:*

*(1) The flow of logic regarding the hypothesis is difficult to understand and needs to be improved.*

*In the fifth paragraph of the introduction, the authors write “… and previous research suggests that age perception may be based on facial surface or shape information (George & Hole, 2000). However, other studies have suggested that facial expression processing relies on holistic processing (Maurer et al., 2002; Tanaka & Sengco, 1997). As shown in previous research, facial expressions interact with other dimensions such as identity and trustworthiness (Engell et al., 2010; Ganel & Goshen-Gottstein, 2002). When the holistic processing of facial expressions is inhibited, observers’ perceptual fields are constricted and facial features are processed sequentially and independently (Rossion, 2009). In such cases, inverted smiling faces could be evaluated as older than upright smiling faces”.*

*Overall, the text in this paragraph seems to be a bit short on explanations and hard to follow. If I understand correctly, I think the authors are trying to say the following here:*

*(a) Some previous studies suggest that age estimation relies on local features of faces.*

*(b) Some previous studies suggest that facial expression processing relies on the holistic processing of the faces.*

*(c) Holistic processing is inhibited when faces are inverted.*

*(d) Disruption of holistic face processing would lead to a greater impact of the local facial feature processing on age estimation. When holistic processing is inhibited, facial expression processing and the related complex cognitive processing would also be inhibited. It has also been reported that facial features are processed sequentially and independently when holistic processing is inhibited.*

*(e) AES may be caused by a local facial feature, that is, wrinkles around the eyes caused by smiling, not by the facial expression of smiling per se.*

*(f) Collectively, due to the relatively heightened impact of local feature processing in inverted faces, inverted smiling faces may be evaluated as older than upright smiling faces.*

*However, the explanation about (e) is lacking from the current text, which makes it difficult to understand the logic here.*

*I also found it confusing that the authors predict/interpret the results of their experiment in terms of “whether holistic processing is involved in AES” in some parts of the manuscript and “whether the relatively heightened impact of local feature processing in the inverted face condition leads to greater AES” in other parts without a clear and explicit distinction between the two.*

**Reply:** The logic of this paragraph is exactly as you have commented. Notably, we have not clarified point (e) in this paragraph. Therefore, we have added a sentence to this paragraph explaining that wrinkles around the eyes drive the AES. We have also revised the paragraphs to enable readers to understand the logic in (a)–(f). Furthermore, based on Reviewer 1's comment, the hypothesis statement has been changed to whether AES is mediated by smiling.

*(2) It is not clear to me why the authors set the surprised face condition. The authors write, “Hence, we set a surprised face (i.e., neutral expression) as the control condition.” in the last paragraph of the introduction. I do not understand this sentence because a surprised face cannot be regarded as an emotionally neutral face and, in addition, the authors set the neutral expression condition besides the surprise expression condition.*

*I assume that the authors wanted to use a facial stimulus with an expression that was not a smile. If so, the authors should describe the difference between smiling and surprised (and neutral) faces in terms of the local features.*

**Reply:** We apologize for this misleading wording. Here the description of “neutral expression” was intended to indicate a neutral emotional valence (neither positive nor negative). The present study used surprised faces because the expression had a change in facial morphology despite neutral emotional valence. Additionally, surprise is a common facial expression used in previous AES studies (Ganel, 2018; Yoshimura et al., 2021). However, we also understand your comment that a surprised face cannot be regarded as emotionally neutral. This is because, for example, in the circumplex affect model, the surprise is neutral in valence but not in arousal (Russell & Barrett, 1999). Moreover, a recent study on facial expressions reported that surprised and neutral faces were mapped separately (Guan, Wei, Hauer, & Liu, 2021). Therefore, we avoided claims about neutral emotions in surprised faces. In our manuscript, we have also explained that surprised faces were selected as facial expression stimuli with facial morphological changes other than smiling.

 Minor concerns and comments:

*(3) Not only the smiling faces but also the surprised faces were estimated to be older in the current experiment. I suspect that this may be due to the wrinkles on the forehead and nasolabial folds when a surprised expression is generated.*

**Reply:** We also suspect that the older age of the surprised face is due to the wrinkles caused by the change in facial expression. This point is included as an interpretation of the results in the Discussion section. Thus, we have revised the paragraph based on your comment (7).

*(4) The last sentence of the third paragraph of the introduction, “These studies indicate that the effect of emotional expressions on age estimation depends on the method of estimation (i.e., directly or retrospectively).”:*

*Does the term “AES” refer only to the phenomenon that smiling faces are estimated to be older than neutral faces in direct age estimations, or does it also refer to the phenomenon that smiling face group is retrospectively estimated to be younger? I got confused to read the sentence above.*

**Reply:** AES refers only to the effect of smiling faces estimated to be older than neutral ones. To avoid this misunderstanding, we explained this phenomenon in the third paragraph.

*(5) The second to last sentence of the fifth paragraph of the introduction, “perceptual fields”:*

*This term requires a brief explanation.*

**Reply:** The description of “perceptual fields” was intended to refer to the scope of a face that observers can process. Therefore, we revised the relevant description in the manuscript to clarify this point.

*(6) Figure 1: The authors should explain what the black dots in this figure are.*

**Reply:** The black dots represent outliers for each facial expression. We added this explanation to the figure.

*(7) The last sentence of the second paragraph of the discussion, “Considering that age is one of the social characteristics critical to encoding the identity of others, the results of this study seem reasonable.”:*

*I am having difficulty figuring out what they mean here. Specifically to which results are they referring?*

**Reply:** This sentence was intended to refer to the result that the age of each expression was perceptually estimated, regardless of the emotional valence of each expression. However, the relevant descriptions are difficult to understand. We also realized that the logical sentence development of this paragraph itself might not convey the point we are trying to argue. Therefore, we revised this paragraph to convey the intent of our argument.

*(8) The first sentence of the fourth paragraph of the discussion, “The findings of the present study indicate that the association between smiling and youth is a misconception, …”:*

*Given that the authors, as they state, did not investigate whether smiling faces are rated as younger in memory-based age estimation, the word “misconception” may be too strong and should be toned down.*

**Reply:** This study is an extension of our previous research (Yoshimura et al., 2021), and we have already investigated whether the association between smiling and youth is a misconception. Therefore, we apologize for the inadequate explanation of this point in the manuscript. Accordingly, we have removed the relevant sections to avoid confusion. Further, we have added sentences to clarify that this study is an extension of the previous studies.

 References

- Ganel T, Goodale MA. The effects of smiling on perceived age defy belief. *Psychon Bull Rev*. 2018;25(2):612–6. http://dx.doi.org/10.3758/s13423-017-1306-8
- Guan H, Wei H, Hauer RJ, Liu P. Facial expressions of Asian people exposed to constructed urban forests: Accuracy validation and variation assessment. *PLoS One*. 2021;16(6):e0253141. http://dx.doi.org/10.1371/journal.pone.0253141
- Russell JA, Barrett LF. Core affect, prototypical emotional episodes, and other things called emotion: Dissecting the elephant. *J Pers Soc Psychol*. 1999;76(5):805–19. https://psycnet.apa.org/fulltext/1999-13561-009.pdf
- Yoshimura N, Morimoto K, Murai M, Kihara Y, Marmolejo-Ramos F, Kubik V, et al. Age of smile: a cross-cultural replication report of Ganel and Goodale (2018). *J Cult Cogn Sci*. 2021;1–15. http://dx.doi.org/10.1007/s41809-020-00072-3

# Peer review: Reviewer response for version 1

**DOI:** 10.5256/f1000research.122814.r134537

**Published:** 2022-05-03

**Authors:** Tzvi Ganel

**Affiliations:** 1 Department of Psychology, Ben-Gurion University of the Negev, Beer-Sheva, Israel

The manuscript is an extension of earlier work that looked at the ageing effect of smiling. This is a nice extension of the effect, which compared it between upright and inverted faces. The AES is maintained in both presentation formats. The authors argue that the results show that age estimations do not rely on holistic processing.

 The research topic is of interest, the manuscript is generally well written, and the paradigm and analyses are solid. However, there are several theoretical reservations that warrant revisions of some of the arguments put forward in the manuscript.

- I feel that the issue of face inversion and its effects on age judgments is not properly discussed. The facial inversion effect refers to the larger decrement in face recognition accuracy due to inversion compared to other objects that are not faces. This effect probably documents holistic, or configural processing of faces. It is true that similar effects have been found for the processing of expression. It is less clear whether age estimations are affected by holistic processing; George & Hole (2000) showed that biases in age perception are similar for upright and inverted faces. ^1^ Yet, the same authors also provided evidence for holistic processing of age, using the composite paradigm (Hole & George, 2011). ^2^ Now, the AES is proposed to be mediated by smile-related wrinkling in the region of the eyes. This effect is probably not based on holistic processing. The current manuscript provides converging evidence for this idea by using face inversion. Therefore, one important note is that the current results do not speak to whether or not age processing is holistic. They do show that the AES is not based on holistic processing.
- To show that age perception is based on holistic processing, one needs to examine accuracy in age perception and to compare it between upright and inverted faces. To compute accuracy, it is crucial to have data about the real ages of the photographed faces and to compute the average absolute error in each condition (Voelkle et al., 2012). ^3^ What the authors do here is computing biases in age estimations. Bias relates to the AES, but bias and accuracy are partially independent variables. A similar amount (and direction) of bias does not necessarily reflect similar accuracy. In order to make arguments about holistic processing of age, the authors need to use the real ages of the faces and to compute accuracy in each condition. Only if accuracy is not substantially impaired for inverted faces, it is possible to argue that age processing is not holistic. Otherwise, the only inference that could be made is that the AES is not based on holistic processing. Such a conclusion is fine, but the authors need to revise the manuscript accordingly.
- In several places in the text, the authors argue that they study “how the holistic processing of facial expressions could be involved in AES”. This assertion is not correct and should be revised. It is true that expression could be processed in a holistic manner, but this doesn’t mean that the effects of smiling on the AES are holistic; first, it is unclear if smiling is processed before age estimations are made. More importantly, the fact that aspects of the smile (e.g., wrinkling) affect age estimation bias cannot be used to infer that the holistic processing of smiling mediates such an effect. The effect is probably mediated by local aspects of the smile rather than by holistic processing. Such arguments in the text should be revised. It is fine to argue that the AES is mediated by smiling rather than to make the unsupported argument that holistic processing is not involved.
- The authors seem to confuse holistic processing with interactive processing between different facial dimensions (e.g., expression and identity). The two aspects could be related to one another, but they are attributed to different mechanisms. The authors should avoid discussing them as the same concept.

Is the work clearly and accurately presented and does it cite the current literature?

Partly

If applicable, is the statistical analysis and its interpretation appropriate?

Yes

Are all the source data underlying the results available to ensure full reproducibility?

Yes

Is the study design appropriate and is the work technically sound?

Yes

Are the conclusions drawn adequately supported by the results?

Partly

Are sufficient details of methods and analysis provided to allow replication by others?

Yes

Reviewer Expertise:

Perception and action; Visual psychophysics; Face perception

I confirm that I have read this submission and believe that I have an appropriate level of expertise to confirm that it is of an acceptable scientific standard, however I have significant reservations, as outlined above.

## References

[1] : The role of spatial and surface cues in the age-processing of unfamiliar faces. *Visual Cognition* .2000;7(4) : 10.1080/135062800394621 485-509 10.1080/135062800394621

[2] : Evidence for holistic processing of facial age. *Visual Cognition* .2011;19(5) : 10.1080/13506285.2011.562076 585-615 10.1080/13506285.2011.562076

[3] : Let me guess how old you are: effects of age, gender, and facial expression on perceptions of age. *Psychol Aging* .2012;27(2) : 10.1037/a0025065 265-77 10.1037/a0025065 21895379

# Response

**Published:** 2022-06-17

**Authors:** Naoto Yoshimura

**Affiliations:** Kyushu University, Japan

*The manuscript is an extension of earlier work that looked at the ageing effect of smiling. This is a nice extension of the effect, which compared it between upright and inverted faces. The* *AES is maintained in both presentation formats. The authors argue that the results show that age estimations do not rely on holistic processing.*

*The research topic is of interest, the manuscript is generally well written, and the paradigm and analyses are solid. However, there are several theoretical reservations that warrant revisions of some of the arguments put forward in the manuscript.*

**Reply:** Thank you for your valuable comment. We are happy to address each comment.

  

- *I feel that the issue of face inversion and its effects on age judgments is not properly discussed. The facial inversion effect refers to the larger decrement in face recognition accuracy due to inversion compared to other objects that are not faces. This effect probably documents holistic, or configural processing of faces. It is true that similar effects have been found for the processing of expression. It is less clear whether age estimations are affected by holistic processing; George & Hole (2000) showed that biases in age perception are similar for upright and inverted faces.1 Yet, the same authors also provided evidence for holistic processing of age, using the composite paradigm (Hole & George, 2011).2 Now, the AES is proposed to be mediated by smile-related wrinkling in the region of the eyes. This effect is probably not based on holistic processing. The current manuscript provides converging evidence for this idea by using face inversion. Therefore, one important note is that the current results do not speak to whether or not age processing is holistic. They do show that the AES is not based on holistic processing.*

**Reply:** As you say, our study does not indicate whether age processing is based on holistic processing, although it could suggest that AES is not based on holistic processing. However, your point is valid, and we are vague in our manuscript regarding this discussion. Therefore, we revised some parts of the manuscript to clarify the argument that the “AES” is not based on holistic processing.

  

- *To show that age perception is based on holistic processing, one needs to examine accuracy in age perception and to compare it between upright and inverted faces. To compute accuracy, it is crucial to have data about the real ages of the photographed faces and to compute the average absolute error in each condition (Voelkle et al., 2012).3 What the authors do here is computing biases in age estimations. Bias relates to the AES, but bias and accuracy are partially independent variables. A similar amount (and direction) of bias does not necessarily reflect similar accuracy. In order to make arguments about holistic processing of age, the authors need to use the real ages of the faces and to compute accuracy in each condition. Only if accuracy is not substantially impaired for inverted faces, it is possible to argue that age processing is not holistic. Otherwise, the only inference that could be made is that the AES is not based on holistic processing. Such a conclusion is fine, but the authors need to revise the manuscript accordingly.*
*　*

**Reply:** Thank you for your suggestion. Unfortunately, as you specified, our study did not examine the accuracy of age perception. This is because the dataset of Japanese stimuli used did not include information on the actual age of each individual. However, the mean absolute error for each condition must be calculated and compared to discuss the holistic processing of age perception. Therefore, we revised the Discussion section to reflect our assertion that AES is not based on holistic processing.

  

- *In several places in the text, the authors argue that they study “how the holistic processing of facial expressions could be involved in AES”. This assertion is not correct and should be revised. It is true that expression could be processed in a holistic manner, but this doesn’t mean that the effects of smiling on the AES are holistic; first, it is unclear if smiling is processed before age estimations are made. More importantly, the fact that aspects of the smile (e.g., wrinkling) affect age estimation bias cannot be used to infer that the holistic processing of smiling mediates such an effect. The effect is probably mediated by local aspects of the smile rather than by holistic processing. Such arguments in the text should be revised. It is fine to argue that the AES is mediated by smiling rather than to make the unsupported argument that holistic processing is not involved.*

**Reply:** We appreciate your pointing this out. Following this comment, we revised the sentences that claim “how the holistic processing of facial expressions could be involved in AES” to those that state “whether the AES is mediated by smiling.”

  

- *The authors seem to confuse holistic processing with interactive processing between different facial dimension (e.g., expression and identity). The two aspects could be related to one another, but they are attributed to different mechanisms. The authors should avoid discussing them as the same concept.*

**Reply:** We appreciate your comment and have removed the description of the facial dimensions and added text about holistic processing.
